# Distinct AMPK-Mediated FAS/HSL Pathway Is Implicated in the Alleviating Effect of Nuciferine on Obesity and Hepatic Steatosis in HFD-Fed Mice

**DOI:** 10.3390/nu14091898

**Published:** 2022-04-30

**Authors:** Hanyuan Xu, Xiaorui Lyu, Xiaonan Guo, Hongbo Yang, Lian Duan, Huijuan Zhu, Hui Pan, Fengying Gong, Linjie Wang

**Affiliations:** Key Laboratory of Endocrinology of National Health Commission, Department of Endocrinology, Peking Union Medical College Hospital, Chinese Academy of Medical Sciences and Peking Union Medical College, Beijing 100730, China; jadef21@foxmail.com (H.X.); lvxiaorui0219@foxmail.com (X.L.); guoxiaonan199708@163.com (X.G.); hongbo.yang7@gmail.com (H.Y.); duanlianpumc@163.com (L.D.); shengxin2004@163.com (H.Z.); panhui20111111@163.com (H.P.)

**Keywords:** Nuciferine (Nuci), AMP-activated protein kinase (AMPK), fatty acid synthase (FAS), hormone sensitive lipase (HSL), obesity, hepatic steatosis

## Abstract

Nuciferine (Nuci), the main aporphine alkaloid component in lotus leaf, was reported to reduce lipid accumulation in vitro. Herein we investigated whether Nuci prevents obesity in high fat diet (HFD)-fed mice and the underlying mechanism in liver/HepG2 hepatocytes and epididymal white adipose tissue (eWAT) /adipocytes. Male C57BL/6J mice were fed with HFD supplemented with Nuci (0.10%) for 12 weeks. We found that Nuci significantly reduced body weight and fat mass, improved glycolipid profiles, and enhanced energy expenditure in HFD-fed mice. Nuci also ameliorated hepatic steatosis and decreased the size of adipocytes. Furthermore, Nuci remarkably promoted the phosphorylation of AMPK, suppressed lipogenesis (SREBP1, FAS, ACC), promoted lipolysis (HSL, ATGL), and increased the expressions of adipokines (FGF21, ZAG) in liver and eWAT. Besides, fatty acid oxidation in liver and thermogenesis in eWAT were also activated by Nuci. Similar results were further observed at cellular level, and these beneficial effects of Nuci in cells were abolished by an effective AMPK inhibitor compound C. In conclusion, Nuci supplementation prevented HFD-induced obesity, attenuated hepatic steatosis, and reduced lipid accumulation in liver/hepatocytes and eWAT/adipocytes through regulating AMPK-mediated FAS/HSL pathway. Our findings provide novel insight into the clinical application of Nuci in treating obesity and related complications.

## 1. Introduction

Obesity exerts much pressure on the public healthcare nationwide [1]. Characterized by excess fat accumulation in adipose and non-adipose tissues, obesity is closely associated with multiple metabolic diseases and contributes to all-cause mortality [2]. Other than surplus food intake, imbalance between lipogenesis, lipolysis, and energy expenditure also exacerbate the deterioration of obesity. Thus, suppressing lipogenesis, inducing lipolysis as well as promoting energy expenditure (EE) would be beneficial in preventing and managing obesity and related morbidities.

Liver and white adipose tissue (WAT) are classic target organs that regulate lipid metabolism. Nonalcoholic steatohepatitis and enlarged visceral WAT, especially epididymal WAT (eWAT) were reported to be notorious obesity comorbidities [3,4]. AMP-activated protein kinase (AMPK), a ubiquitously distributed serine/threonine protein kinase which is involved in a spectrum of metabolic processes plays a vital role in energy metabolism including lipid synthesis and catabolism in both liver and eWAT [5]. It was reported that AMPK inhibited de novo fat synthesis by regulating the expressions of transcription factor sterol-regulatory element binding protein 1 (SREBP1) and its downstream rate-limiting enzymes including fatty acid synthase (FAS) and acetyl-CoA carboxylase (ACC) [6]. Lipolysis was mainly catalyzed by hormone-sensitive lipase (HSL) and adipose triglyceride lipase (ATGL) [7]. Studies have also shown that the AMPK phosphorylated Ser406 of ATGL to stimulate TG hydrolase activity and activate lipolysis [8]. In hepatocytes, a central catabolism process, fatty acid oxidation, is delicately regulated by key transcription factor peroxisome proliferator-activated receptor α (PPARα) and its downstream enzyme carnitine palmitoyltransferase 1α (CPT1α) [9]. In adipocytes, there is another catabolism mechanism where stored fat can be decomposed to generate heat, and the best effector that characterized this non-shivering thermogenesis is uncoupling protein 1 (UCP1) [10]. Activating UCP1 in WAT to develop thermogenic capacity remain a promising approach for enhancing energy expenditure thus ameliorating obesity [11]. Besides adipokines that are secreted primarily in adipose tissue, fibroblast growth factor 21 (FGF21) [12,13] and zinc-α2-glycoprotein (ZAG) [14] were also reported to positively serve the whole-body metabolism targeting liver, eWAT, and other tissues. To date, both thermogenesis and the expressions of FGF21 and ZAG were also found to be associated with the AMPK pathway [15,16].

In decades, many anti-obesity drugs have been developed, yet their use was banned due to severe side effects [17]. In traditional Chinese medicine, there are various botanic products derived from natural plants used for treating obesity [18,19], and they have been increasingly studied by the modern medical sciences since less side effects were observed. Among these products, lotus leaf (Nelumbo nucifera leaf) embraces a long history of treating obesity and related complications. Nuciferine (Nuci), the main aporphine alkaloid component in lotus leaf, were found to possess beneficial effects on inflammation [20], cardiovascular diseases [21], and cancer [22]. Our previous study revealed that in vitro, Nuci is also capable of inhibiting proliferation and differentiation of preadipocytes [23], suggesting that it may exhibit an anti-obesity effect. In 2018, Zhang C, et al. reported that Nuci may act through a PPARα/PPARγ coactivator-1α pathway to ameliorate hepatic steatosis [24]. Another in vivo report found that Nuci decreased the lipid droplets and promoted the expression of glucose transporter type 4 (GLUT-4) in mature 3T3-L1 adipocytes with possible involvement of activation of AMPK [25]. However, full awareness of the underlying molecular mechanism remains lacking. Therefore, in this study, enlightened by previous reports, we aimed at exploring the alleviating effect of Nuci on obesity and related hepatic steatosis, and at investigating the role of AMPK in Nuci-induced decrease of lipid accumulation in both liver and eWAT in HFD-fed mice. Another highlight of this study is that to our knowledge, this is the first report of Nuci promoting energy expenditure in HFD-fed mice, further shedding light on another potential mechanism by which the anti-obesity effect of Nuci occurs.

## 2. Materials and Methods

### 2.1. Animal Experiments

Nuci (purity ≥ 98% by HPLC, CAS: 475-83-2) was purchased from Biopurify Phytochemicals Ltd. (Chengdu, China). The molecular weight of Nuci is 295.38. Six-week-old C57BL/6J male mice were purchased from HFK Bioscience Co., Ltd. (Beijing, China) and housed in a standard facility with 12 h dark/light cycle (3 mice/cage). After 7 days acclimation, the animals were randomly allocated to four groups. Mice in the control group were fed a normal diet (ND, 10% kcal fat, AIN93M, Trophic Animal Feed High-Tech Co., Ltd., Nantong, China; *n* = 12). Mice in the high-fat diet (HFD) group were fed a HFD (45% kcal fat, D12451, Trophic Animal Feed High-Tech Co., Ltd., Nantong, China; *n* = 12); mice in the HFD-Nuci group (HFD-Nuci) were fed a HFD supplemented with 0.10% Nuci (*n* = 12). The customized feeds were made by a commercial company from where the regular feeds were bought. According to the manufacturer of the experimental diets (Trophic Animal Feed High-Tech Co., Ltd., Nantong, China), the Nuci in powder form was first completely dissolved in ethanol and mixed with the diets at room temperature until it is uniformly distributed. Then the ethanol was evaporated by adequate air blowing evaporation, and the mixture was then made in the form of bar as regular diet for experimental application. Moreover, liraglutide, a glucagon like peptide-1 (GLP-1) receptor agonists approved for the treatment of obesity [26] was used as reference treatment. Mice in the HFD-liraglutide group (HFD-Lira) were fed an HFD with daily subcutaneous injection of liraglutide (15676, Novo Nordisk, Bagsværd, Denmark) at 200 μg/kg (*n* = 12). Mice in the ND, HFD, and HFD-Nuci groups received daily subcutaneous administration with equal volume of saline as controls compared to the HFD-Lira group. Mice in the ND, HFD, HFD-Lira groups were fed with standard ND/HFD, respectively, without Nuci supplementation as controls to the HFD-Nuci group. Body weight and food intake of each mouse were recorded twice a week. After treatment of 12 weeks, intraperitoneal glucose tolerance test (IPGTT) and intraperitoneal insulin tolerance test (IPITT) were conducted, then the 48-h energy expenditure was monitored by Promethion Metabolic Cage System (Sable Systems, North Las Vegas, NV, USA). At the end of the experiment, animals were fasted overnight (12 h) and sacrificed with CO_2_ gas. Blood samples were collected for biochemical detections including liver function markers ALT and AST, lipid metabolism-related traits including total cholesterol (TC), triglycerides (TG), high density lipoprotein-cholesterol (HDL-C), low density lipoprotein-cholesterol (LDL-C), fasting blood glucose (FBG), and fasting serum insulin (FINS). Insulin resistance (HOMA-IR) indexes were calculated as HOMA-IR = FBG × FINS/22.5. Liver, brown adipose tissue (BAT), and white adipose tissue (WAT) including eWAT, inguinal WAT (iWAT), and perirenal WAT (pWAT) were collected, weighed, and immediately frozen in liquid nitrogen and stored at −80 °C for molecular experiments. A bulk of the liver and eWAT were fixed in 4% paraformaldehyde for histology observation; another portion of liver from each mouse was placed in optimum cutting temperature (OCT) medium (Sakura Finetek Co., Ltd., Torrance, CA, USA) and stored at −80 °C.

All experiments conducted on animals were approved by the Medical Ethics Committee of Peking Union Medical College (XHDW-2019-010, Beijing, China) and followed by the National Institutes of Health regulations regarding animal care and use (Beijing, China).

### 2.2. Indirect Calorimetry

Indirect calorimetry was conducted by Promethion Metabolic Cage System in accordance with the manufacturer’s instructions. Mice were acclimated to the chambers for 24 h before the measurements began. Then the animals were kept under 12 h day/night cycle (from 8:00 a.m. to 8:00 p.m.) at 24 °C with feeding regimen described in Materials and Methods 2.1Day and night parameters of thermogenesis including O_2_ consumption (VO_2_), CO_2_ consumption (VCO_2_), energy expenditure (EE), and home-cage locomotor activity were measured in the following 48 h. VO_2_, VCO_2_, and EE were normalized against total body weight of each mouse. Respiratory exchange ratio (RER) was calculated as the ratio of VCO_2_ versus VO_2_.

### 2.3. Histology of Liver and eWAT

Livers and eWATs were fixed in 4% paraformaldehyde, then dehydrated and embedded in paraffin. About 3 μm thick slides of tissue were obtained and stained using hematoxylin and eosin (H&E) according to the standard protocol. Histological images were obtained using a light microscope with digital camera (Nikon eclipse Ti, Nikon Corporation, Tokyo, Japan) and a stereoscopic microscope (BX53, Olympus Optical Co., Ltd., Tokyo, Japan) equipped with a charged-coupled device (CCD) camera (DP80; Olympus) at 200× and 400× magnifications, respectively. 

Furthermore, H&E stained slides of eWAT were further used to determine the diameters of each adipocyte in the field (400×) using Image J software (Olympus). Next, six fields of H&E stained slides of liver were chosen randomly for measurement per mouse. Liver characteristics of non-alcoholic fatty liver disease (NAFLD) were quantified by a NAFLD scoring system for rodent as described previously [27]. Briefly, the scoring system consists of steatosis score and inflammation score. For steatosis scores, there are three individual features including macrovesicular steatosis, microvesicular steatosis, and hypertrophy. Macrovesicular and microvesicular steatosis were scored based on the percentage of the total affected area: 0 (<5%), 1 (5–33%), 2 (34–66%), and 3 (>66%). The difference between macrovesicular and microvesicular steatosis was quantified by the position of the nucleus, i.e., the vacuoles displace the nucleus sideways (macrovesicular) or not (microvesicular). Hepatocellular hypertrophy was merely defined as cellular enlargement more than 1.5 times the normal hepatocyte diameter (distinct from ballooning). The level of hypertrophy was also scored based on the percentage of the affected area: 0 (<5%), 1 (5–33%), 2 (34–66%), and 3 (>66%). For inflammation scores, it was evaluated by counting the number of inflammatory foci per field. Five different fields were counted, and the average was subsequently scored into the following categories: 0 (<0.5 foci), 1 (0.5–1.0 foci), 2 (1.0–2.0 foci), 3 (>2.0 foci). The sum of the NAFLD scores ranged from 0~12, the greater the number, the more severe the condition of steatosis. These sections were read by the same pathologist in a blind manner.

For Oil-red O staining of frozen sections of liver, the frozen sections of liver were prepared in 8 μm thickness and stained with freshly diluted and filtered Oil Red O dye (Sigma, St. Louis, MO, USA). Hematoxylin staining was used as counterstaining. Samples were observed and photographed by a stereoscopic microscope (BX53, Olympus Optical Co, Ltd., Japan) equipped with a charged-coupled device (CCD) camera (DP80; Olympus) at magnification of 200×.

### 2.4. IPGTT and IPITT 

IPITT and IPGTT were performed at the 10th week of intervention. In IPGTT, mice were fasted for 12 h and intraperitoneal administration of 50% glucose (2 g/kg) was conducted. In IPITT, mice were fasted for 6-h and intraperitoneal administration of insulin (0.4 IU/kg for the ND group, 0.5 IU/kg for HFD-fed groups) (Novolin R, Novo Nordisk, Denmark) was conducted. Blood glucose from caudal vein were measured at 0, 30, 60, 90, 120 min after the injections. Blood glucose curve were drawn and area under curve (AUC) of glucose was calculated accordingly. 

### 2.5. Cell Culture of 3T3-L1 Preadipocytes and HepG2 Hepatocytes

The 3T3-L1 preadipocytes and HepG2 hepatocytes were cultured in accordance with previously used protocols in our laboratory [28]. In brief, 3T3-L1 cells were cultured in DMEM/F12 medium, mixed with 10% fetal bovine serum (FBS) and 1% Penicillin-Streptomycin (Hyclone, Logan, UT, USA) at 37 °C under 5% CO_2_. The HepG2 hepatocytes were cultured in minimum essential medium with Earle’s Balanced Salts (Hyclone, Logan, USA) supplemented with 1% penicillin-streptomycin and 0.1 mM non-essential amino acids (Solarbio Life Sciences, Beijing, China) at 37 °C under 5% CO_2_.

### 2.6. Cell Isolation and Culture of Human Primary Preadipocytes and Murine Primary Mature Adipocytes 

Human primary preadipocytes were obtained from the visceral WAT harvested during laparoscopic sleeve gastrectomy of a 21-year-old obese female (BMI = 35.5 kg/m^2^). The comorbidities of the visceral WAT donor include insulin resistance, polycystic ovarian syndrome (PCOS), hypertension (grade 1, low risk), and chronic superficial gastritis and atopic dermatitis. The study was approved by the Medical Ethics Committee of Peking Union Medical College (No. JS-1093), and an informed consent was given to the patient before the surgery. Preadipocytes was isolated and cultured as previously described in our published paper [23]. Briefly, approximately 8 g of human visceral fat pad was harvested and digested by the same volume of type I collagenase (2 mg/mL) (Life Technologies, Van Allen Way, CA, USA). Then digestion was stopped by same volume of basal culture medium consisting of DMEM/F12 medium, 1% penicillin-streptomycin, 10% FBS, 17 μM pantothenic acid, 10 μg/mL transferrin, and 33 μM biotin (Sigma, St. Louis, MO, USA). The mixture was filtered and centrifuged at 600× *g* for 5 min. Sediment at the bottom of the tube was re-suspended and cultured in a cell culture flask (T25, Corning, NY, USA) at 37 °C under 5% CO_2_. 

The murine primary mature adipocytes were isolated from bilateral eWAT of 4 mice in each group by the “celling culture” method [29]. In brief, 0.6~2.0 g murine eWAT was digested by type I collagenase, and the digestion was terminated by the same volume of basal medium. Then the mixture was filtered and centrifuged. The floating mature adipocytes were re-suspended with culture medium and seeded in a T25 filled with basal medium. The flask was turned upside down and incubated at 37 °C under 5% CO_2_ for 48 h. Then the culture medium was gently poured, and the flask was turned back and filled with 5 mL culture medium and continued incubating at 37 °C under 5% CO_2_. Images of adipocytes were captured by a digital camera ((Nikon eclipse Ti, Nikon Corporation, Japan) at 100× magnification.

### 2.7. Cell Viability Assay

Effects of palmitic acid (PA) and Nuci of different concentrations on HepG2 hepatocytes, 3T3-L1 or human primary preadipocytes viability were determined by Cell Count Kit-8 (CCK-8, MedChem Express, HY-K0301, Monmouth Junction, NJ, USA). Cells were seeded in 96-well plates with a density of 1 × 10^3^/well. After 24 h, cells were treated with 0, 2.5, 5, 10, 20 μM Nuci for 48 h, or with 0, 0.2, 0.4, 0.6, 0.8 mM PA for 12 h. Cell medium was then changed to fresh culture medium and mixed with 10 μL 2-(methoxy-4-nitrophenyl)-3-(4-nitrophenyl)-5-(2,4-disulfophenyl)2H-tetrazolium (WST-8) solution for each well and incubated for 4 h. Optical density (OD) values were measured at a wavelength of 450 nm by a spectrophotometer (Thermo, Waltham, MA, USA).

### 2.8. Cell Experiments of HepG2 Hepatocytes

HepG2 hepatocytes were cultured as described in Section 2.5. Nuci was dissolved in vehicle (dimethyl sulfoxide, DMSO) (Solarbio Life Sciences, China) for cell experiments. The maximum concentration of DMSO is 0.5%. After cells achieved 70~80% of confluence, medium was changed to fresh culture medium containing 0, 2.5, 5, 10, 20 μM Nuci. About 48 h later, cells were harvested and lysed for further molecular experiments. 

### 2.9. Cell Experiments for AMPK Activities in Adipocytes and HepG2 Hepatocytes

3T3-L1 preadipocytes and primary human preadipocytes were induced differentiation as previously described [23]. Briefly, after 48 h of confluence, cells were cultured in differentiation medium supplemented with 10 μM dexamethasone, 10 μg/mL insulin, and 0.5 mM 3-isobutyl-1-methylxanthine (IBMX) (Gibco BRL, Grand Island, NY, USA) for 4 days. Then the medium was changed to fresh culture medium supplemented with 10 μg/mL insulin for another 2 days. Subsequently, the culture medium was refreshed every 2 days. Fully differentiated adipocytes were divided into five groups. The control group was treated with vehicle, the Nuci groups were treated with either 10 or 20 μM Nuci for 48 h, and the AMPK inhibitor Compound C (CC) (MedChem Express, USA, HY-13418A) groups were pretreated with 10 μM CC for 1 h, followed by treatment with either 10 or 20 μM Nuci for 48 h.

For HepG2 hepatocytes, 0.4 mM palmitic acid (PA) was used to induce lipid accumulation. Cells were divided into six groups. The control group was treated with vehicle, the PA group was treated with PA (0.4 mM) for 12 h. The Nuci groups were treated with either 10 or 20 μM Nuci for 48 h and together with PA for the last 12 h. The CC groups were pretreated with 10 μM CC for 1 h, followed by treatment with either 10 or 20 μM Nuci for 48 h and together with PA for the last 12 h.

### 2.10. Oil Red O Staining and TG Contents Determination in Adipocytes and HepG2 Hepatocytes

The experiment procedures were described in our published paper [30]. In brief, cells were stained with freshly diluted and filtered Oil Red O dye (Sigma, St. Louis, MO, USA) for 2 h. Representative photographs were captured by a digital camera (Nikon) at 100× magnification. Oil red O dye was extracted by isopropanol and the OD value was measured at 492 nm. TG contents were measured using a commercial kit (Comin Biotechnology, Suzhou, China). Briefly, cells were disrupted using an ultrasonic cell-crushing machine (Q700, Qsonica, Newtown, CT, USA). OD values were then measured by a spectrometer at 505 nm and the intracellular lipid contents were normalized against total protein concentration.

### 2.11. Real-Time Quantitative PCR 

Real-time quantitative PCR (RT-qPCR) was performed in accordance with previous description [28]. In brief, the expression of housekeeping gene PPIA [31], lipid metabolism-related genes (FAS, ACC, SREBP1, HSL, ATGL), fatty acid oxidation-related genes (PPARα, CPT1α), adipokine genes (FGF21, ZAG), and thermogenesis gene (UCP1) were measured using a PCR system (ABI7500, Applied Biosystems, Bedford, MA, USA). Primer sequences were listed in Supplementary Table S1. The relative expressions of target genes were calculated by the 2^−^^△△Ct^ method [32].

### 2.12. Simple Western Analysis 

Total protein content was extracted by RIPA lysis buffer containing Protease and Phosphatase Inhibitor Cocktail (MedChem Express, USA) and measured using a BCA Protein Assay Reagent kit (Beyotime Biotechnology, Shanghai, China). Protein expressions were detected by Simple Western using a Size Separation Master Kit with Split buffer (12–230 kD) following the instructions of the manufacturer. The reliability of this experimental approach has been verified by previous studies [33,34]. In brief, 1.5 μg protein of each sample was separated by capillary electrophoresis on Wes instrument (Protein Simple, San Jose, CA, USA) under default settings. Protein levels were probed with rabbit anti-AMPK (1:50, #2532, Cell Signaling Technology, Danvers, MA, USA), rabbit anti-phospho-AMPK (Thr172) (1:50, #2535, Cell Signaling Technology, USA), and rabbit anti-β-actin (13E5) (1:50, #4970, Cell Signaling Technology, USA). An Anti-Rabbit Detection Module was purchased from Protein Simple. Data were analyzed by Compass software (Protein Simple, San Jose, CA, USA) version 5.0.1.

### 2.13. Plasmid Transfection and Dual Luciferase Reporter Assay System

PGL3-hFAS (−622 ~ +3 bp)-Luc (hFAS625-Luc) plasmid which contains human FAS promoter from −622 ~ +3 bp [23] and PGL3-hHSL (−697 ~ +53 bp)-Luc (hHSL750-Luc) plasmid which contains human HSL promoter from −697 ~ +53 bp [30] were previously constructed in our laboratory. In brief, hFAS625-Luc, hHSL750-Luc plasmids and their internal control plasmid pRL-SV40 were transiently transfected into the cells, and 0~20 μM Nuci was added for 48 h. Both firefly and renilla luciferase activities were measured by a Dual-Luciferase Reporter Assay System Kit (Promega, Madison, WI, USA) in an automated optical immunoassay analyzer (Beijing Pilot Biotechnology Corporation, Beijing, China). Each concentration was repeated for 9~12 wells.

### 2.14. Statical Analyses

Cells experiments were carried out independently for at least three times. Results were expressed as mean ± standard error (SE). Statical analyses were performed by SPSS software for Windows (version 25.0) (SPSS Inc., Chicago, IL, USA). Chi square test, one-way and two-way ANOVA were used in data analyses. The Kruskal–Wallis test was used if ANOVA was not applicable. For post-hoc test, the Dunnett *t* test (two-side) or Dunnett t3 post-test was used for comparisons between three or more groups. *p* < 0.05 was considered as statistically significant in all analyses.

## 3. Results

### 3.1. Nuci Prevented Obesity in HFD-Fed Mice

**Figure 1:**
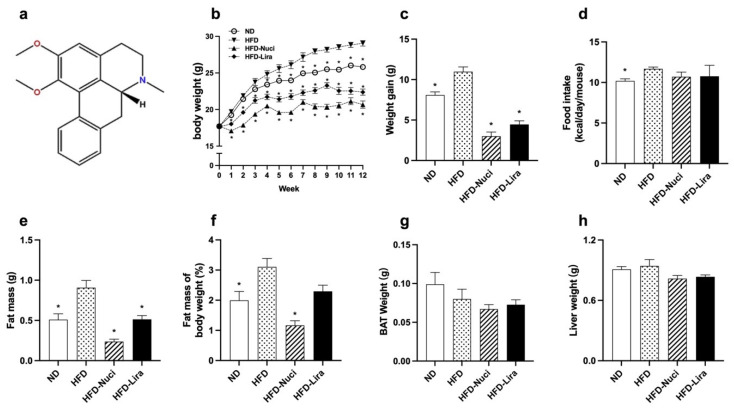
Nuci prevented obesity in HFD-fed mice. (**a**) The molecular structure of Nuci. Seven-weeks male C57BL/6J mice were fed with ND, HFD, HFD supplemented with Nuci and HFD with subcutaneous injection of Lira at 200 μg/kg/day for 12 weeks, body weight (**b**) and energy intake (**d**) were recorded, and body weight gain (**c**) was calculated by the end of the experiment. The amount of fat mass (**e**), including sWAT, eWAT, and pWAT, as well as the weight of BAT (**g**) and liver (**h**) were recorded. Fat mass of body weight (**f**) was calculated by dividing the sum of fat mass by body weight. Data were presented as mean ± SE, *n* = 12 in each group. * *p* < 0.05 vs. the HFD group.

The molecular structure of Nuci was displayed in Figure 1a. As shown in Figure 1b,c, compared with the ND group, the body weight and body weight gain of the HFD group were remarkedly increased from the fourth week till the last. Treatment of Nuci remarkably reduced the body weight of mice at the first week, with the maximal reduction effect being observed by the end of the experiment, where the body weight of mice decreased by 28.8% compared to the HFD group. Similarly, Lira remarkedly decreased body weight since the first week, with the maximal reduction effect being 23.0% less than the HFD group. Both Nuci and Lira effectively impeded the HFD-induced weight gain by 72.6% and 59.4% (*p* < 0.05). As shown in Figure 1d, HFD significantly elevated energy intake compared to the ND, yet Nuci and Lira had little effect on energy intake in HFD-fed mice.

As depicted in Figure 1e,f, HFD remarkedly increased the amount of fat mass (including sWAT, eWAT and pWAT), while Nuci notably decreased fat mass and fat mass percentage by 73.8% and 58.7% compared with the HFD group (*p* < 0.05). Lira exhibited similar effect (*p* < 0.05). There was no effect of Nuci or Lira on the weight of BAT or liver (Figure 1g,h).

### 3.2. Nuci Improved Dyslipidemia, Glucose Tolerance and Insulin Resistance in HFD-Fed Mice

**Figure 2:**
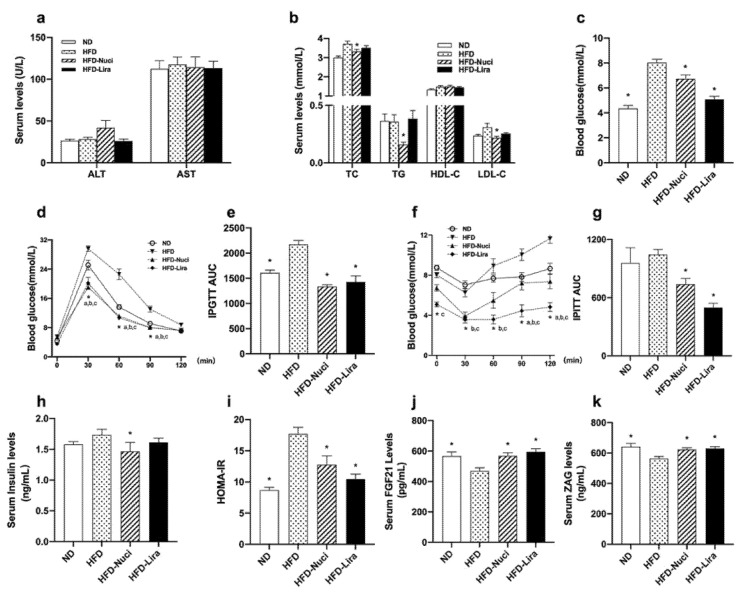
Nuci improved dyslipidemia, glucose tolerance, and insulin resistance in HFD-fed mice. Seven-weeks male C57BL/6J mice were fed with ND, HFD, HFD supplemented with Nuci and HFD with subcutaneous injection of Lira at 200 μg/kg/day for 12 weeks. Liver function markers including ALT and AST (**a**), lipid metabolism-related traits including TC, TG HDL-C, and LDL-C (**b**) and fasting blood glucose (**c**) were examined. The intraperitoneal glucose tolerance test (IPGTT, (**d**)) and the intraperitoneal insulin tolerance test (IPITT, (**f**)) were performed at the 10th week, and area under curves (AUCs) of IPGTT (**e**) and IPITT (**g**) were calculated. FINS (**h**) was determined, and HOMA-IR (**i**) was calculated accordingly. Serum levels of FGF21 (**j**) and ZAG (**k**) were determined by ELISA. Data were presented as mean ± SE, *n* = 12 in each group. * *p* < 0.05 vs. the HFD group. In Figure 2d and f, * a, the ND vs. the HFD group; * b the HFD-Nuci vs. the HFD group; * c the HFD-Lira vs. the HFD group.

Liver function remained similar among the groups (Figure 2a). As shown in Figure 2b, Nuci remarkedly decreased serum levels of TC, TG, and LDL-C by 10.6%, 55.9%, and 30.1%, respectively, when compared with the HFD group (all *p* < 0.05). As shown in Figure 2c, Nuci and Lira notably decreased FBG levels of HFD-fed mice (*p* < 0.05). IPGTT and IPITT were performed (Figure 2d and f, *p* < 0.05) and as depicted in Figure 2e,g, Nuci and Lira reduced AUC of IPGTT and IPITT compared with the HFD group, respectively (*p* < 0.05). Nuci also effectively improved insulin sensitivity as reflected by decreased FINS levels and HOMA-IR. The hypoglycemic effect of Nuci was similar to Lira (Figure 2h,i, *p* < 0.05). Next, serum levels of FGF21 and ZAG were measured. As shown in Figure 2j,k, HFD led to a notable decrease in both adipokines compared to the ND group (*p* < 0.05), whereas the serum levels of which were remarkedly increased to the similar content as the ND group after treatment of Nuci or Lira (*p* < 0.05). 

### 3.3. Nuci Remarkedly Increased Energy Metabolism in HFD-Fed Mice 

**Figure 3:**
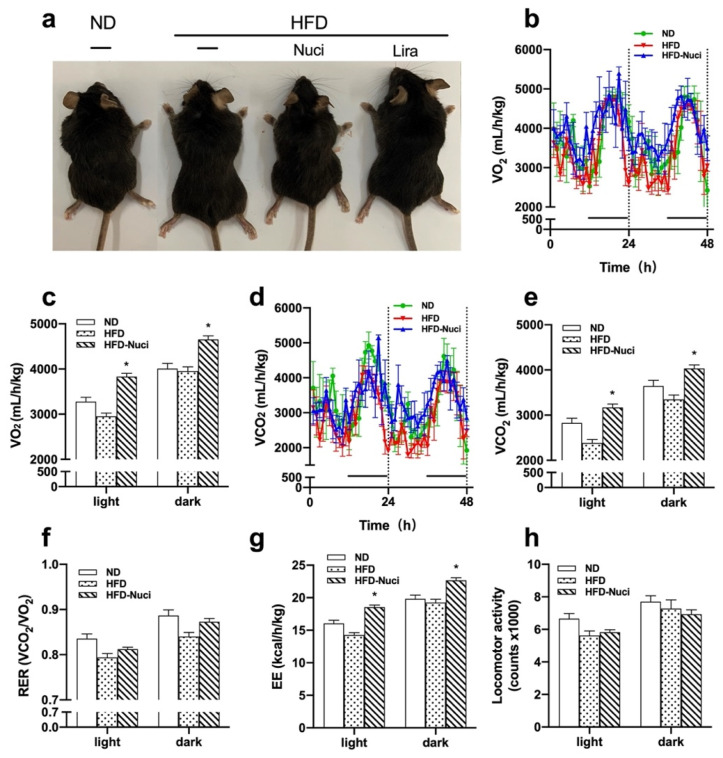
Nuci increased energy metabolism in HFD-fed mice. Seven-weeks male C57BL/6J mice were fed with ND, HFD, HFD supplemented with Nuci and HFD with subcutaneous injection of Lira at 200 μg/kg/day for 12 weeks. General pictures of mice in ND, HFD, HFD-Nuci, and HFD-Lira group were presented (**a**). After 11 weeks of treatment, mice in the ND, HFD, and HFD-Nuci group were placed in metabolic cages at 22 °C at 12 h light–dark cycle. VO_2_ (**b**), VCO_2_ (**d**) were detected and quantification of VO_2_ (**c**), VCO_2_ (**e**), RER (**f**), EE (**g**), and locomotor activity (**h**) during both the light and dark cycle were determined by Promethion. Data were presented as mean ± SE, *n* = 4 in each group. * *p* < 0.05 vs. the HFD group.

As shown in Figure 3a, the representative general pictures of mice showed that the HFD-fed mouse was larger than the ND-fed ones and the treatment of Nuci made them comparatively smaller. Next, the effect of Nuci on energy metabolism including VO_2_, VCO_2_, RER, and locomotor activity levels were measured in metabolic cages. As depicted in Figure 3b,c, Nuci remarkedly elevated VO_2_ consumption, especially in the light cycle where the VO_2_ of HFD-Nuci group increased to 1.33-fold of the HFD group (*p* < 0.05). Similar effect was observed in VCO_2_ (Figure 3d,e). RER was not remarkedly altered by Nuci treatment (Figure 3f). Moreover, it was observed that Nuci significantly increased EE to 1.41-fold in the light cycle and 1.17-fold in the dark cycle in comparison with the HFD group, respectively (Figure 3g, *p* < 0.05). However, the locomotor activity reflected by beam breaks showed no statistically significant difference among the three groups (Figure 3h). 

### 3.4. Nuci Ameliorated Hepatic Steatosis by Activating AMPK Phosphorylation in Liver of HFD-Fed Mice

**Figure 4:**
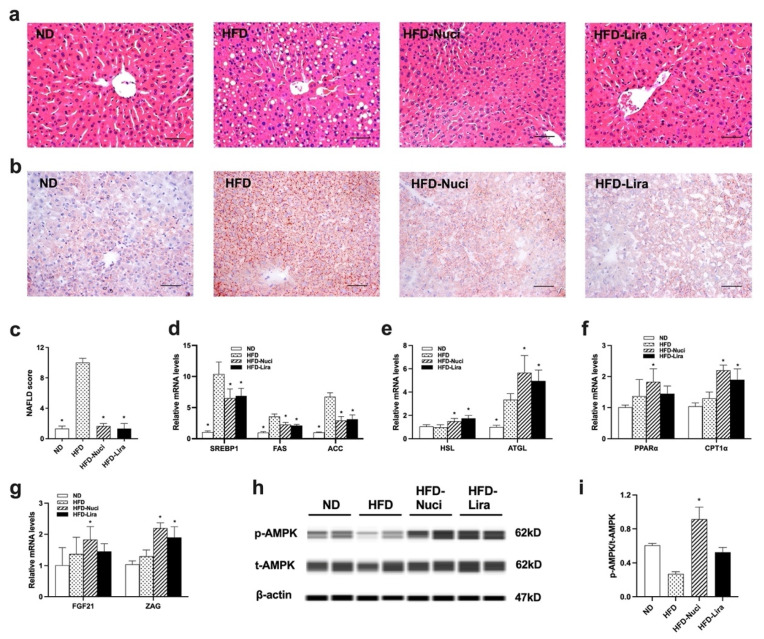
Nuci ameliorated hepatic steatosis by activating AMPK phosphorylation in liver of HFD-fed mice. Seven-weeks male C57BL/6J mice were fed with ND, HFD, HFD supplemented with Nuci and HFD with subcutaneous injection of Lira at 200 μg/kg/day for 12 weeks. H&E-staining (**a**) and Oil-red O staining (**b**) images of representative sections of mice liver in the ND, HFD, HFD-Nuci, and HFD-Lira group; photographs were taken at 200× magnification. NAFLD scores of the four groups according to a general NAFLD scoring system for rodent models (**c**). The mRNA expressions of lipogenesis-related genes SREBP1, FAS, and ACC (**d**), lipolysis-related genes HSL and ATGL (**e**), fatty acid oxidation-related genes PPARα and CPT1 (**f**), and adipokines FGF21 and ZAG (**g**) were determined by RT-qPCR. The protein levels of p-AMPK, t-AMPK and β-actin were analyzed by Simple western (**h**). the expression of p-AMPK was normalized against t-AMPK (**i**). Data were presented as mean ± SE, *n* = 12 in each group. * *p* < 0.05 vs. the HFD group.

As shown in Figure 4a,c, the histology of liver by H&E staining in HFD-fed mice presented obvious steatosis, indicated by distinct hepatocellular ballooning and parenchymal steatosis. The NAFLD scores also remarkedly increased in the HFD group. However, Nuci and Lira remarkedly decreased NAFLD scores when compared with the HFD group (*p* < 0.05). Moreover, as shown in Figure 4b, the Oil-red O staining also revealed that the liver of mice in the HFD group showed greater amount of stained lipid deposition compared to that in the ND group, indicating that HFD feeding induced significant hepatic steatosis. After treatment of Nuci and Lira, the hepatic lipid accumulation was ameliorated as evidenced by reduced Oil-red O-stained lipid droplets in livers of mice in the HFD-Nuci and HFD-Lira group. Next, as depicted in Figure 4d, HFD-fed mice presented substantially elevated expressions in lipogenesis-related genes including SREBP1, FAS, and ACC to 9.67-, 3.62-, and 6.64-fold of the ND group, respectively, and Nuci and Lira remarkably reduced the expressions of these genes (*p* < 0.05). Besides, they also remarkably increased the expressions of HSL and ATGL (Figure 4e, *p* <0.05), as well as PPARα and CPT1α (Figure 4f, *p* < 0.05). Interestingly, the expressions of FGF21 and ZAG in liver were also prominently increased to 1.82- and 2.20-fold of HFD group by Nuci treatment, and the expression of ZAG also increased upon Lira treatment (Figure 4g, *p* < 0.05). Western blot revealed that HFD feeding remarkedly downregulated the phosphorylation of AMPK in liver, while Nuci treatment remarkedly increased the p-AMPK upregulation by 3.38-fold in liver when compared to the HFD group (Figure 4h,i, *p* < 0.05).

### 3.5. Nuci Suppressed Lipid Accumulation by Reducing Lipogenesis and Promoting Lipolysis in HepG2 Hepatocytes

**Figure 5:**
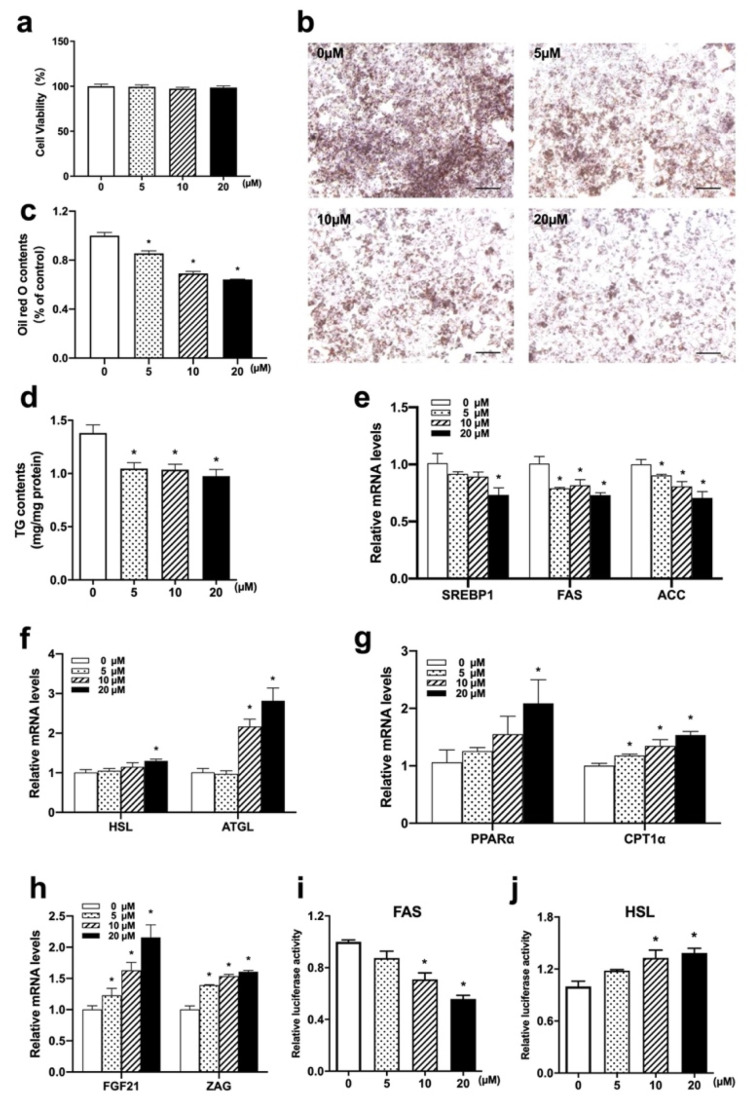
Nuci suppressed lipid accumulation by reducing lipogenesis and promoting lipolysis in HepG2 hepatocyte. Cell viability after treatment of 0~20 μM Nuci were determined by CCK-8 (**a**). Cells were stained by Oil red O and photographed at 100× magnification (**b**) and Oil red O dye was extracted and detected (**c**). Intracellular TG contents (**d**) were determined by a commercial kit following the manufacturer’s instructions. The mRNA expressions of lipogenesis-related genes SREBP1, FAS, and ACC (**e**), lipolysis-related genes HSL and ATGL (**f**), fatty acid oxidation-related genes PPARα and CPT1 (**g**), and adipokines FGF21 and ZAG (**h**) were determined by RT-qPCR. Cells were transiently transfected with hFAS625-Luc and hHSL750-Luc plasmids and the luciferase activities of FAS (**i**) and HSL (**j**) were measured. The firefly luciferase activities were adjusted by the renilla luciferase. Data were presented with mean ± SE from three independent experiments, each concentration was repeated for 9~12 wells. * *p* < 0.05 vs. the control group (0 μM).

HepG2 hepatocytes were treated with 0, 10, 20 μM Nuci for 48 h after achieving 70–80% confluence. As shown in Figure 5a, the viability of HepG2 hepatocytes treated with 5~20 μM Nuci yielded between 97.3~101.1%, suggesting Nuci of applied concentrations did not elicit cell toxicity. Next, as shown in Figure 5b,c, 5~20 μM Nuci dose-dependently decreased the intracellular lipid contents; the maximal effect was observed at 20 μM where the lipid contents were reduced by 35.8% compared to the control group (0 μM) (*p* < 0.05). Similarly, TG contents were also remarkedly decreased (Figure 5d, *p* < 0.05). Moreover, similar to the results obtained in liver tissue, 20 μM Nuci remarkedly suppressed the expressions of SREBP1, FAS, and ACC by 27.7%, 27.0%, and 29.4%, respectively (Figure 5e, *p* < 0.05), while promoted the expressions of HSL, ATGL, PPARα, and CPT1α (Figure 5f,g, *p* < 0.05). Moreover, the expressions of FGF21 and ZAG were also promoted by Nuci with the maximal effect observed at 20 μM, where they were increased to 2.16- and 1.61-fold of the control group (0 μM), respectively (Figure 5h, *p* < 0.05). In order to further investigate the underlying mechanism by which Nuci stably regulates the expression of FAS and HSL, hFAS625-Luc and hHSL750-Luc plasmids were transiently transfected into HepG2 s cells and treated with Nuci for 48 h. It was found that the 20 μM Nuci notably suppressed the luciferase activities of FAS by 44.2%, while promoted HSL to 1.39-fold of the control group (0 μM) (Figure 5i,j, *p* < 0.05). 

### 3.6. Nuci Prevented PA-Induced Cellular Steatosis by Activating AMPK Phosphorylation in HepG2 Hepatocytes

**Figure 6:**
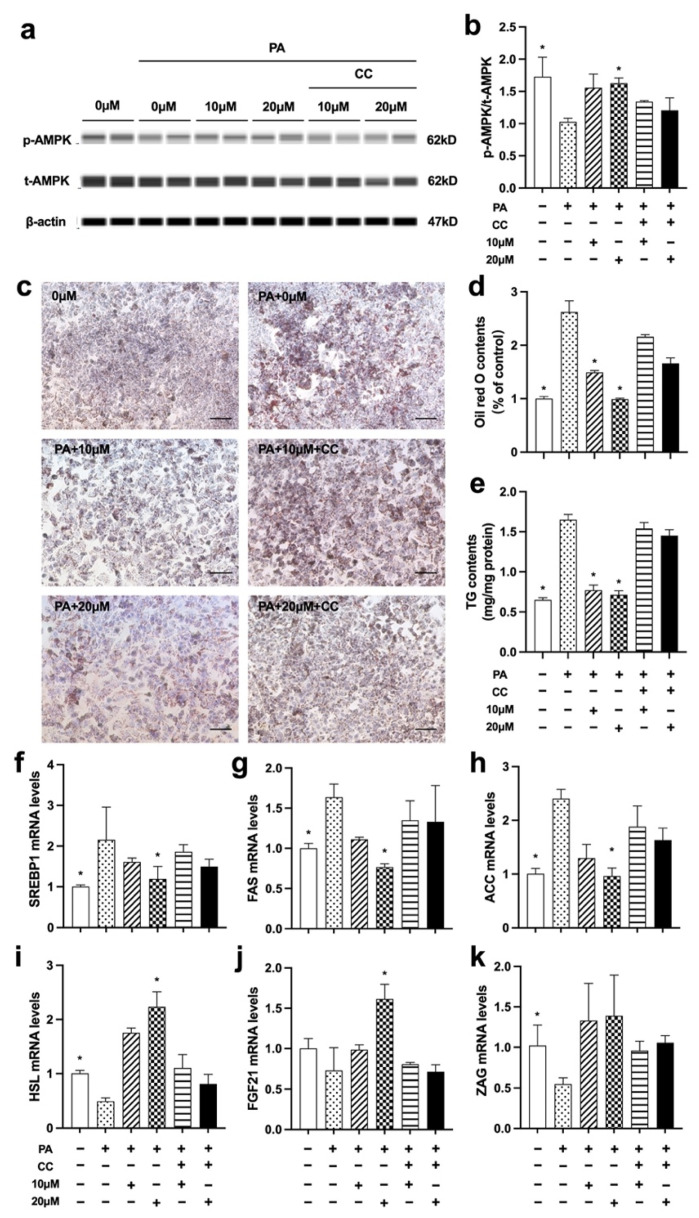
Nuci prevented PA-induced cellular steatosis by activating AMPK phosphorylation in HepG2 hepatocytes. HepG2 hepatocytes were cultured and treated according to the scheme described in Method 2.9. The protein levels of p-AMPK, t-AMPK, and β-actin were analyzed by Simple western (**a**), the expression of p-AMPK was normalized against t-AMPK (**b**). Oil red O staining was conducted and photographed at 100× magnification (**c**) and Oil red O dye was extracted and detected (**d**). Intracellular TG contents I were determined by a commercial kit following the manufacturer’s instructions. The mRNA expressions of SREBP1 (**f**), FAS (**g**), ACC (**h**), HSL (**i**), ZAG (**j**), and FGF21 (**k**) were determined by RT-qPCR. Data were presented with mean ± SE from three independent experiments, * *p* < 0.05 vs. the PA group (PA + 0 μM).

As remarkedly elevated AMPK phosphorylation was observed in liver of HFD-Nuci group, we further deployed PA-induced steatosis cell model to investigate whether AMPK was involved in alleviating the effect of Nuci on lipid accumulation in PA-induced HepG2 cells. As shown in Figure 6a–e, PA notably decreased AMPK phosphorylation while remarkedly increased intracellular lipid droplets and TG contents in HepG2 cells (*p* < 0.05). However, 10 and 20 μM Nuci restored the AMPK phosphorylation and ameliorated the PA-induced lipid accumulation. Next, a selective AMPK inhibitor CC was used to further confirm the role of AMPK. The result showed that CC remarkedly abolished the promotion of AMPK phosphorylation induced by Nuci (Figure 6b). Consequently, the prevention of lipid accumulation caused by Nuci was notably impeded in the presence of CC as demonstrated by the deepened Oil red O staining (Figure 6c), the increased lipid contents (Figure 6d), and intracellular TG contents (Figure 6e). Similarly, as shown in Figure 6f–k, the reduced expressions of SREBP1, FAS, and ACC and the promoted expression of HSL and FGF21 caused by 20 μM Nuci were also notably impeded after CC pretreatment (*p* < 0.05). All these findings suggested that Nuci suppressed lipogenesis-related genes and promoted lipolysis gene via activating AMPK signaling pathway in HepG2 hepatocytes. 

### 3.7. Nuci Decreased the Weight of eWAT by Activating AMPK Phosphorylation in HFD-Fed Mice

**Figure 7:**
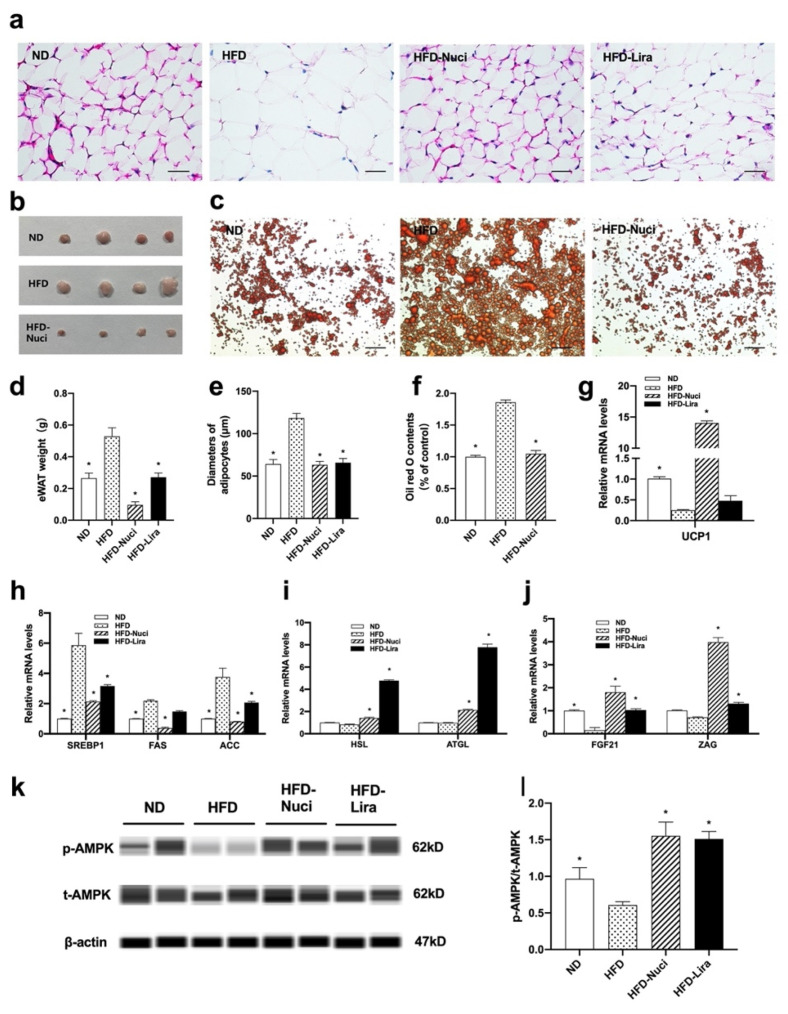
Nuci decreased the weight of eWAT by activating AMPK phosphorylation in HFD-fed mice. Seven-weeks male C57BL/6J mice were fed with ND, HFD, HFD supplemented with Nuci and HFD with subcutaneous injection of Lira at 200 μg/kg/day for 12 weeks. H&E-staining images of representative sections of mice eWAT in the ND, HFD, HFD-Nuci, and HFD-Lira group, photographs were taken at 400× magnification (**a**). Diameters of adipocytes were measured from six random fields using Image J software (**e**). The weight of eWAT was measured (**d**). Photographs of isolated mice eWATs in the ND, HFD, HFD-Nuci group (**b**). Representative images of the Oil red O stained mice primary mature adipocytes, photographs were taken at 100× magnification (**c**) and Oil red O dye was extracted and detected (**f**). The mRNA expressions of UCP1 (**g**) lipogenesis-related genes SREBP1, FAS, and ACC (**h**), lipolysis-related genes HSL and ATGL (**i**), adipokines FGF21 and ZAG (**j**) were determined by RT-qPCR. The protein levels of p-AMPK, t-AMPK, and β-actin were analyzed by Simple western (**k**), the expression of p-AMPK was normalized against t-AMPK (**l**). Data were presented as mean ± SE, *n* = 12 in each group. * *p* < 0.05 vs. the HFD group.

eWATs in the ND, HFD, HFD-Nuci group were harvested and photographed (Figure 7b). As shown in Figure 7d, both Nuci and Lira remarkedly decreased the weight of eWAT in HFD-fed mice (*p* < 0.05). Moreover, HFD-fed mice presented remarkedly enlarged adipocytes as demonstrated by H&E staining, while Nuci and Lira inhibited the morbid expansion of adipocytes to the extent where it is much alike to the mice fed with ND (Figure 7a,e, *p* < 0.05). As shown in Figure 7c,f, Oil red O staining of primary mature adipocytes in eWATs revealed that the size and number of lipid droplets were remarkedly enlarged in the HFD group, whereas Nuci decreased the lipid contents by 43.2% compared to the HFD group (*p* < 0.05). Notably, HFD led to significant downregulation of UCP1, a vital marker for non-shivering thermogenesis, whereas Nuci completely blocked this phenomenon and strongly promoted the mRNA levels of UCP1 in eWAT to 14.0-fold of that in the HFD group (Figure 7g, *p* < 0.05). Likewise, it was shown that Nuci remarkably reduced the mRNA levels of SREBP1, FAS, and ACC by 63.7%, 82.6%, and 78.5%, respectively (Figure 7h, *p* < 0.05), and promoted the mRNA levels of HSL, ATGL (Figure 7i, *p* < 0.05), FGF21, and ZAG (Figure 7j, *p* < 0.05). Lira showed similar effects on the expressions of these genes. Further mechanical study demonstrated that the phosphorylation of AMPK was remarkably decreased in HFD group when compared to ND group as shown in Figure 7k,l, and the treatment of both Nuci and Lira remarkedly increased the p-AMPK/t-AMPK ratio to 2.55- and 2.48-fold of HFD group, respectively (*p* < 0.05).

### 3.8. Nuci Inhibited Lipid Accumulation by Activating AMPK Phosphorylation in Fully Differentiated 3T3-L1 Cells 

**Figure 8:**
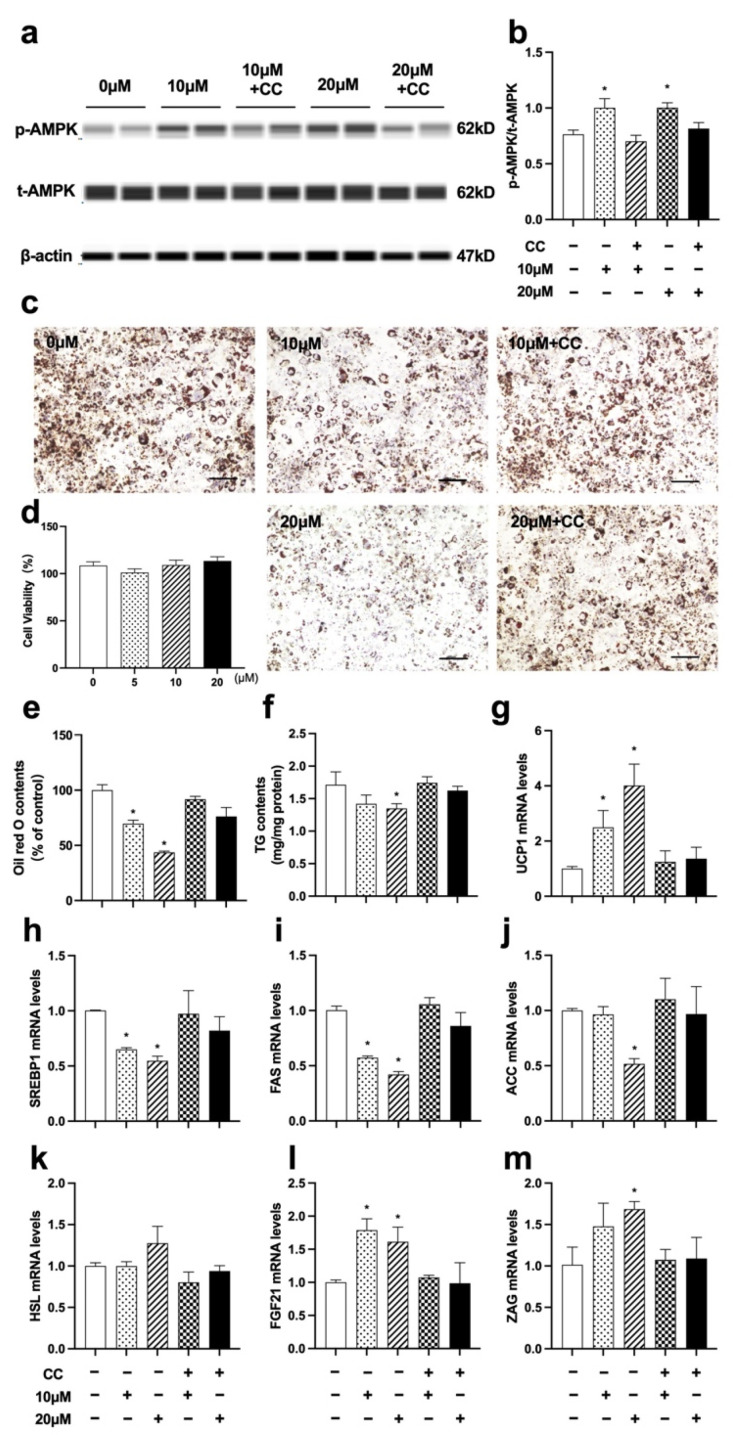
Nuci inhibited lipid accumulation by activating AMPK phosphorylation in fully differentiated 3T3-L1 adipocytes. 3T3-L1 preadipocytes were cultured and induced differentiation, then fully differentiated adipocytes were treated as described in the Method 2.9. The protein levels of p-AMPK, t-AMPK, and β-actin were analyzed by Simple western (**a**), the expression of p-AMPK was normalized against t-AMPK (**b**). Oil red O staining was conducted and photographed at 100× magnification (**c**) and Oil red O dye was extracted and detected (**e**). Cell viability after treatment of 0~20 μM Nuci were determined by CCK-8 (**d**). Intracellular TG contents (**f**) were determined by a commercial kit following the manufacturer’s instructions. The mRNA expressions of UCP1 (**g**), SREBP1 (**h**), FAS (**i**), ACC (**j**), HSL (**k**), FGF21 (**l**), and ZAG (**m**) were determined by RT-qPCR. Data were presented with mean ± SE from three independent experiments, * *p* < 0.05 vs. the control group (0 μM).

Since the remarkedly elevated AMPK phosphorylation was observed in eWAT of HFD-Nuci group, the causal relationship between the increased phosphorylation of AMPK and the fat loss remained unclear. In order to answer this question, we further explore the effect of Nuci on phosphorylation of AMPK and lipid accumulation in fully differentiated 3T3-L1 cells. Similar to Result 3.5, first, the viability of 3T3-L1 adipocytes was confirmed to be unaffected upon administration of 5~20 μM Nuci (93.5~104.6%) (Figure 8d). Next, as shown in Figure 8a,b, 10 and 20 μM Nuci directly and remarkedly increased the phosphorylation of AMPK, with this effect being abolished after pretreatment of CC. Subsequently, 10 and 20 μM Nuci directly decreased the lipid contents by 30.3% and 56.4%, respectively, as demonstrated by Oil red O staining, and this effect was also remarkedly abolished with CC (Figure 8c,e, *p* < 0.05). Similar results were observed in TG contents measurements (Figure 8f, *p* < 0.05). Furthermore, 10~20 μM Nuci directly and notably decreased the expressions of SREBP1, FAS, and ACC by 35.0~58.0% (Figure 8h–j, *p* < 0.05), while promoted the expressions of FGF21, ZAG as well as UCP1, and these effects being blocked by CC as well (Figure 8g,I,m, *p* < 0.05). Together, these findings suggested that Nuci directly and remarkedly reduced intracellular lipid accumulation through regulating AMPK-mediated FAS/HSL pathway and promoting FGF21, ZAG as well as UCP1 expression.

### 3.9. Nuci Inhibited Lipid Accumulation by Activating AMPK Phosphorylation in Fully Differentiated Human Primary Adipocytes 

**Figure 9:**
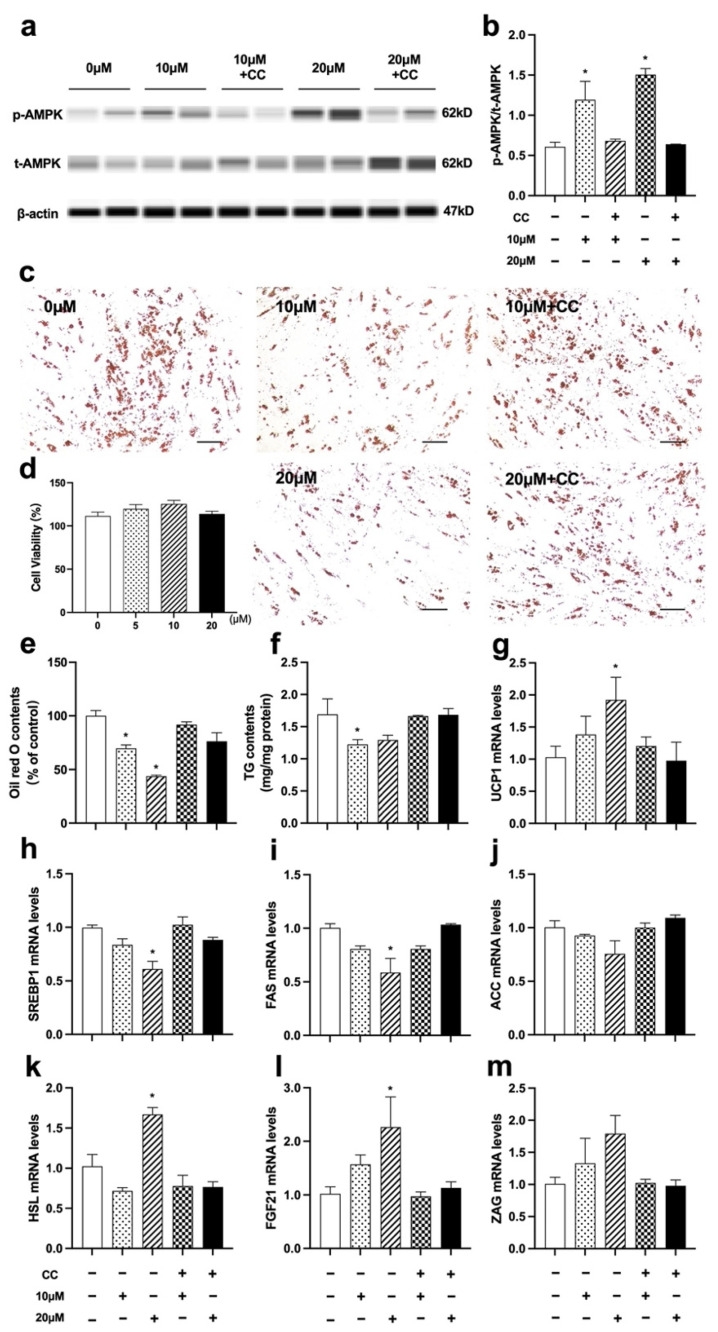
Nuci inhibited lipid accumulation by activating AMPK phosphorylation in fully differentiated human primary adipocytes. Human primary preadipocytes were cultured and induced differentiation, then fully differentiated adipocytes were treated as described in the Method 2.9. The protein levels of p-AMPK, t-AMPK, and β-actin were analyzed by Simple western (**a**), the expression of p-AMPK was normalized against t-AMPK (**b**). Oil red O staining was conducted and photographed at 100× magnification (**c**) and Oil red O dye was extracted and detected (**e**). Cell viability after treatment of 0~20 μM Nuci was determined by CCK-8 (**d**). Intracellular TG contents (**f**) were determined by a commercial kit following the manufacturer’s instructions. The mRNA expressions of UCP1 (**g**), SREBP1 (**h**), FAS (**i**), ACC (**j**), HSL (**k**), FGF21 (**l**), and ZAG (**m**) were determined by RT-qPCR. Data were presented with mean ± SE from three independent experiments, * *p* < 0.05 vs. the control group (0 μM).

Primary cells have the additional advantages of being unmodified, which is reputed to reflect in vivo conditions more closely than cell lines. Therefore, we harvested primary preadipocytes from human visceral WAT to further investigate the beneficial role of Nuci. As expected, 0~20 μM Nuci did not alter cell viability in human primary adipocytes (103.9~125.5%) (Figure 9d). As shown in Figure 9a,b, 20 μM Nuci remarkedly boosted AMPK phosphorylation by 1.31-fold in the control group (0 μM) (*p* < 0.05). In the meantime, intracellular lipid contents were also decreased by Nuci (Figure 9c,e,f, *p* < 0.05). Similar to Results 3.8, CC remarkedly decreased the phosphorylation of AMPK, led to the subsequent inhibition of lipid accumulation induced by Nuci rendered ineffective. Furthermore, as demonstrated in Figure 9g–m, 20 μM Nuci notably decreased the expressions of SREBP1 and FAS by 39.0% and 41.3%, while increased the mRNA levels of UCP1, HSL, and FGF21 to 1.92, 1.67, 2.26-fold of the control group, respectively (*p* < 0.05), with all these effects being reversed by CC.

## 4. Discussion

Recently, herbal medicines have attracted much attention due to their potential for easy availability, less side effects, and wide application. Nuci, as the main component of lotus leaf, have been reported with anti-obesity effect on murine models, yet the mechanism remains controversial. Thus, in this study, we demonstrated that Nuci remarkedly prevented HFD-induced weight gain and improved glycolipid metabolism. It was also worth noting that for the first time, we reported that Nuci increased energy expenditure in HFD-fed mice. Besides, Nuci also ameliorated hepatic steatosis and decreased weight and size of eWAT. In hepatocytes and adipocytes, Nuci was found to suppress lipogenesis, promote lipolysis by directly regulating a AMPK-mediated FAS/HSL pathway, and increased fatty acid oxidation, thermogenesis, and the expressions of adipokines including FGF21 and ZAG. 

As indicated by glucose indicators including IPGTT, IPITT, and HOMA-IR, Nuci restored HFD-induced impairment of glucose tolerance and insulin sensitivity similar to that of the positive control drug liraglutide. In consistence with our results, several studies reported that Nuci notably prevented obesity and improved glucose metabolism in HFD-fed diabetic mice [24,35]. Furthermore, Nuci also improved plasma lipid profiles without affecting liver function, indicating that the anti-obesity effect of Nuci did not attribute to toxicity. This finding was supported by Guo F et al. who observed similar results in HFD-fed hamsters treated with Nuci [36]. One striking finding of our present study is that Nuci was first found to notably increase VO_2_, VCO_2_, and EE in HFD-fed mice without altering locomotor activity or energy intake. It is well established that the increase of EE can be further attributed to the increase of RER or basal metabolic rate [37]. Since there were no significant differences found in RER between the groups in the present study, it is more likely that Nuci prevents obesity in HFD-fed mice, at least partly, via enhancing EE, and the increase of EE was thought to be associated with the reinforced basal metabolic rate. 

Excessive weight gain is often accompanied by hepatic steatosis and morbid expansion of adipose tissue. In the present study, we found that HFD-fed mice exhibit severe ballooning degeneration in liver, while Nuci ameliorated the development of HFD-induced hepatic steatosis. Consistently, Wang et al. also found that 8 weeks of Nuci supplementation achieved similar effects in HFD-fed rats [38]. Besides, in this study, Nuci also remarkedly reduced the amount of eWAT, deflated the volume of adipocytes, and decreased the intracellular lipid contents. There were studies that reported that Nuci decreased the weight and protected against histological degeneration of visceral WAT induced by high-fat diet in hamsters and mice [35,36], yet the mechanism was not thoroughly investigated. In the present study, Nuci supplementation remarkedly decreased the mRNA levels of lipogenesis-related transcription factors including SREBP1 and its target genes FAS and ACC, upregulated the expressions of key enzymes in lipolysis including HSL and ATGL in both liver and eWAT. Similarly, Guo F et al. revealed that 8 weeks of Nuci downregulated hepatic expressions of lipogenesis-related genes in HFD-fed hamsters [36]. Enlightened by these results, we further conducted in vitro experiments and observed comparable effects of Nuci in HepG2 hepatocytes, 3T3-L1 adipocytes, and human primary adipocytes, confirming that Nuci directly modulate lipid metabolism at cellular levels. Moreover, for the first time, we found that 0~20 μM Nuci remarkedly inhibited FAS and promoted HSL promoter activities in HepG2 cells, suggesting that Nuci may suppress lipogenesis and activate lipolysis by regulating the FAS/HSL pathway.

Fatty acid oxidation contributes to lipid catabolism as well, especially in the liver. Herein, we found that the expressions of a critical transcription factor in fatty acid oxidation, PPARα, as well as its downstream gene CPT1α in liver were boosted by Nuci. In consistence with our finding, Guo F et al. also revealed that in HFD-fed hamsters, Nuci had protective effects on hepatic lipid metabolism by promoting PPARα and CPT1α [36]. Zhang et al. established HFD/STZ-induced diabetic mice and observed similar phenomenon; further molecular experiments showed that the binding of activated PPARα to PPRE was reinforced by Nuci [24]. Moreover, in vitro experiments in the present study further confirmed that Nuci promoted the expression of PPARα and CPT1α in hepatocytes, indicating that Nuci alleviated fat accumulation by promoting fatty acid oxidation in liver and hepatocytes. 

Previous studies have shed light on the involvement of adipokines in lipid mobilization in metabolic organs, among which FGF21 and ZAG possessed renowned regulatory effect on fat metabolism [14,39]. It has been reported that FGF21 suppressed hepatic lipogenesis in an autocrine and paracrine manner [40], and overexpressing ZAG promoted lipolysis and fatty acid oxidation in hepatocytes and boosted browning in adipocytes [41]. Our previous work demonstrated that Nuci promoted the expression of FGF21 and ZAG in adipocytes [23], and in this study, we found for the first time that in vivo, Nuci also promoted the expression of these two adipokines in both liver and eWAT, leading to higher levels of serum FGF21 and ZAG. Together, Nuci may reduce fat accumulation by enhancing the expression of FGF21 and ZAG, which further exerted their beneficial role on glycolipid metabolism in an endocrine manner. Moreover, browning of WAT is emerging as a potential therapeutic target for weight loss. When stimulated, WAT acquires a brown fat-like phenotype, leading to increased thermogenesis through dissipating energy as heat [42]. Notably, we first demonstrated that Nuci led to dramatic elevation in mRNA levels of the key non-shivering thermogenesis activator UCP1 in eWAT. Likewise, Nuci also promoted the mRNA levels of UCP1 in fully differentiated adipocytes derived from 3T3-L1 and human primary preadipocytes, suggesting Nuci may promote browning of WAT by directly increasing the expression of UCP1 in HFD-fed mice. It also provides an explanation that the enhanced EE induced by Nuci in HFD-fed mice might be resulting from stimulating eWAT browning, thus promoting thermogenesis and increasing basal metabolic rate. 

The AMPK pathway is crucial for maintaining cellular homeostasis, especially for lipid metabolism. Activation of AMPK leads to weight loss and amelioration of NAFLD [43]. Ma C et al. demonstrated that Nuci activated AMPK phosphorylation and inhibited lipogenesis in insulin-resistant 3T3-L1 adipocytes [25], yet the in vivo situation remained unclear. In the present study, we first demonstrated that in HFD-fed mice, AMPK pathway was involved in the beneficial role of Nuci in preventing HFD-induced obesity and attenuating hepatic steatosis. First, HFD remarkedly decreased AMPK activity, while Nuci significantly restored AMPK activity to the extent similar to that of ND group in both liver and eWAT. Second, cell experiment performed in hepatocytes and adipocytes showed that Nuci directly and remarkedly increased the phosphorylation of AMPK, and this effect was abolished after pretreatment of CC. Finally, the Nuci-induced suppression of lipid accumulation, which was derived from the decreased lipogenesis (SREBP1, FAS and ACC), increased lipolysis (HSL), as well as fatty acid oxidation (PPARα and CPT1α) or thermogenesis (UCP1), were all notably impeded after inhibition of AMPK. A previous study showed that AMPK is an upstream kinase of SREBP-1c which directly inhibits its nuclear translocation [44], hereby suppressing downstream lipogenesis. Furthermore, the activation of AMPK also increases lipolysis and fatty acid oxidation [15], and upregulated the expression of adipokine FGF21 levels [45]. Consistently, in this study, the elevation of FGF21 and ZAG mRNA levels induced by Nuci in hepatocytes and adipocytes was also abolished by CC, indicating that AMPK may be involved in the effect of Nuci on regulating the expression of both adipokines. All these findings suggest that Nuci improves whole body glucose and lipid metabolism and ameliorates hepatic steatosis through directly activating AMPK signaling pathway, which subsequently leads to the inhibition of lipogenesis, promotion of lipolysis and fatty acid oxidation, as well as upregulation of the expressions of FGF21 and ZAG in liver/hepatocytes and eWAT/adipocytes. Still, how Nuci acts with the blockage of AMPK pathway in HFD-induced obesity mice was not confirmed, hence further in vivo studies centering on Nuci and AMPK pathway are needed.

Moreover, it should be noted that different from our study, there are research that reported Nuci may also exert its anti-obesity effect via regulation of gut microbiota. Gut microbiota is essential for maintaining normal gastrointestinal and immune functions and efficient digestion of nutrients [46], and increasingly abundant evidence has shown that the gut microbiota affects whole-body nutrient acquisition, energy regulation, and fat storage [47]. Pharmacokinetics studies of Nuci showed that the absolute bioavailability is relatively low (1.9 ± 0.8%), yet mild dosage of Nuci was reported to show robust anti-obesity effect. Therefore, it is reasonable to speculate that apart from the ability of regulating the expressions of lipid-metabolism genes, the metabolic benefits of Nuci may also be related to the regulation of gut microbiota. Wang et al. [38] found that Nuci significantly reduced the body weight of HFD-fed rats after 8 weeks of intervention, 16S rRNA sequencing revealed that it decreased the ratio of Firmicutes/Bacteroidetes, the relative abundance of the LPS-producing Desulfovibrio, and other bacteria involved in lipid metabolism, whereas it increased the relative abundance of short-chain fatty acids (SCFA)-producing bacteria, enhanced intestinal integrity, leading to lower blood endotoxemia to reduce inflammation in HFD-fed rats. Consistent with this result, Yu et al. [48] also found that Nuci reduced body weight in HFD-fed mice. By applying metagenomic sequencing, it was found that Nuci significantly changed the construction of gut microbiota, enriched the abundance of a potential probiotic Akkermansia muciniphila. However, the anti-obesity effect of Nuci was abolished after pretreatment of antibiotics, suggesting that gut microbiota plays an important role in the metabolic benefits of Nuci. Similarly, Tang et al. [35] found that Nuci corrected intestinal dysbacteriosis in gestational diabetic mice, enriched probiotic abundances of Akkermansia, Lactobacillus and Bifidobacterium, while reduced conditional pathogen abundances including Escherichia-Shigella and Staphylococcus. Additionally, the study of Shi et al. [47] found that Nuci reduced body weight gain and fat accumulation in HFD-fed mice by improving intestinal permeability and autophagy [49].

## 5. Conclusions

To summarize, the present study illustrated that Nuci prevented obesity, improved glycolipid metabolism, attenuated hepatic steatosis and enhanced energy expenditure in HFD-fed mice. These beneficial role of Nuci was closely associated with the AMPK-mediated inhibition of lipogenesis and promotion of lipolysis, together with upregulation of adipokines, fatty acid oxidation, and thermogenesis in liver/hepatocytes and eWAT/adipocytes. Overall, our results show for the first time that AMPK-mediated FAS/HSL pathway was implicated in the beneficial effect of Nuci in reducing lipid accumulation, providing experimental evidence for clinical application of Nuci in treating obesity and related metabolic dysfunctions.

## Data Availability

The corresponding authors are responsible for data availability on reasonable request.

## Supplementary Materials

The following are available online at https://www.mdpi.com/article/10.3390/nu14091898/s1. Table S1: Primers used for RT-qPCR analysis.
