# Supplementary material for: Distinct AMPK-Mediated FAS/HSL Pathway Is Implicated in the Alleviating Effect of Nuciferine on Obesity and Hepatic Steatosis in HFD-Fed Mice
Source: Nutrients. 2022 Apr 30;14(9):1898. doi: 10.3390/nu14091898 (PMC9101490; doi:10.3390/nu14091898)
Supplement: Supplementary file 1 [file nutrients-14-01898-s001.zip › nutrients-1666715-supplementary.pdf]

Supplementary materials

1 Supplementary table

Table S1. Primers used for RT-qPCR analysis

**Gene**	**Forward primer**	**Reverse primer**
m-FAS	GGAGGTGGTGATAGCCGGTAT	TGGGTAATCCATAGAGCCCAG
m-FGF21	CTGCTGGGGGTCTACCAAG	CTGCGCCTACCACTGTTCC
m-ACC	GATGAACCATCTCCGTTGGC	GACCCAATTATGAATCGGGAGTG
m-SREBP1	TGACCCGGCTATTCCGTGA	CTGGGCTGAGCAATACAGTTC
m-PPIA	GAGCTGTTTGCAGACAAAGTTC	CCCTGGCACATGAATCCTGG
m-ZAG	AGCAAAGGTTTTCCGAGGTTT	GAGACCCTGTAGTGTCCTTGTAA
m-HSL	CCAGCCTGAGGGCTTACTG	CTCCATTGACTGTGACATCTCG
m-ATGL	TTCACCATCCGCTTGTTG	AGTTCCACCTGCTCAGAC
m-PPARα	AGAGCCCCATCTGTCCTCTC	ACTGGTAGTCTGCAAAACCAAA
m-CPT1α	AACAACGGCAGAGCAGAG	CCACATAGAGGCAGAAGAGG
m-UCP-1	TCTCTGCCAGGACAGTACCCAA	GAGTCGCAGAAAAGAAGCCACAA
h-FAS	AAGGACCTGTCTAGGTTTGATGC	TGGCTTCATAGGTGACTTCCA
h-FGF21	ATGGATCGCTCCACTTTGACC	GGGCTTCGGACTGGTAAACAT
h-ACC	ATGTCTGGCTTGCACCTAGTA	CCCCAAAGCGAGTAACAAATTCT
h-SREBP1	TGGCTGCTCAATGGGCTGTT	GCGATGCCTCCAGAAGTACACG
h-PPIA	CCCACCGTGTTCTTCGACATT	GGACCCGTATGCTTTAGGATGA
h-ZAG	GCTTACCTGGAGGAGGAGTG	TTCCCTGGGTAGAAGTCGTAG
h-HSL	CTCTGGTCTACTACGCCCAG	CATCCCTTATGCAGCGTGAC
h-ATGL	ATGGTGGCATTTCAGACAACC	CGGACAGATGTCACTCTCGC
h-PPARα	CCAGTATTTAGGAAGCTGTCCTG	CGTTGTGTGACATCCCGACAG
h-CPT1α	TCCAGTTGGCTTATCGTGGTG	TCCAGAGTCCGATTGATTTTTGC

Note: m: mouse; h: human
